# Chylous ascites associated with intestinal obstruction from volvulus due to Petersen’s hernia: report of a case

**DOI:** 10.1186/s40792-016-0207-9

**Published:** 2016-07-28

**Authors:** Yuichi Akama, Tetsuya Shimizu, Itsuo Fujita, Yoshikazu Kanazawa, Daisuke Kakinuma, Hitoshi Kanno, Aya Yamagishi, Hiroki Arai, Eiji Uchida

**Affiliations:** Department of Gastrointestinal and Hepato-Biliary-Pancreatic Surgery, Nippon Medical School Hospital, 1-1-5 Sendagi, Bunkyo-Ku, Tokyo Japan

**Keywords:** Chylous ascites, Intestinal obstruction, Ischemia

## Abstract

**Background:**

Chylous ascites is an uncommon finding which is usually associated with recent abdominal/oncologic or retroperitoneal surgery. It is not usually seen in cases of acute obstruction.

**Case presentation:**

A patient who had previously undergone a laparoscopy-assisted distal gastrectomy with Roux-en-Y reconstruction for early gastric cancer presented with acute abdominal pain and epigastric fullness. Computed tomography suggested small bowel obstruction due to volvulus. We were able to reduce the volvulus and close a Petersen’s hernia without resecting the bowel; a large amount of chylous ascites was an incidental finding.

**Conclusions:**

We present a case of chylous ascites occurring in a setting of small bowel obstruction due to Petersen’s hernia, 3 years after successful distal gastrectomy for early gastric cancer, with no evidence of tumor recurrence.

## Background

A large amount of ascites can accompany bowel obstruction with nonviable bowel loops [1]. Ascites is occasionally bloody [2] and can be seen in obstruction cases requiring intestinal resection. Chylous ascites, however, has not been commonly observed with bowel obstruction. Here, we report a case of a large amount of chylous ascites associated with small bowel obstruction from a Petersen’s hernia-associated volvulus, which was successfully treated without bowel resection.

## Case presentation

An 85-year-old man presented to our hospital with a 5-h history of abdominal pain and distention. He attended the outpatient department at our hospital for regular checkups but had been asymptomatic until he visited out emergency department. At age 82 years, he had undergone laparoscopy-assisted distal gastrectomy with Roux-en-Y reconstruction for early gastric cancer. An antecolic isoperistaltic gastrojejunostomy was performed using an endoscopic linear stapler. A serosubmucosal single-layer hand-sewn anastomosis (Jourdan’s) was performed through a small abdominal incision for Y reconstruction. The mesentery of the Y loop was closed using nonabsorbable braided polyester (3-0) interrupted sutures, while the Petersen’s defect was not closed. Pathology revealed a stage IB (T2N0M0) tumor; the resection stumps were negative. His postoperative course had been unremarkable. Computed tomography (CT) performed 1.5 months prior to admission showed no evidence of tumor recurrence and no ascites. The patient had no other relevant medical history.

Physical examination on arrival showed focal tenderness and distention in the epigastrium. His body temperature was 36.4 °C.

**Fig. 1:**
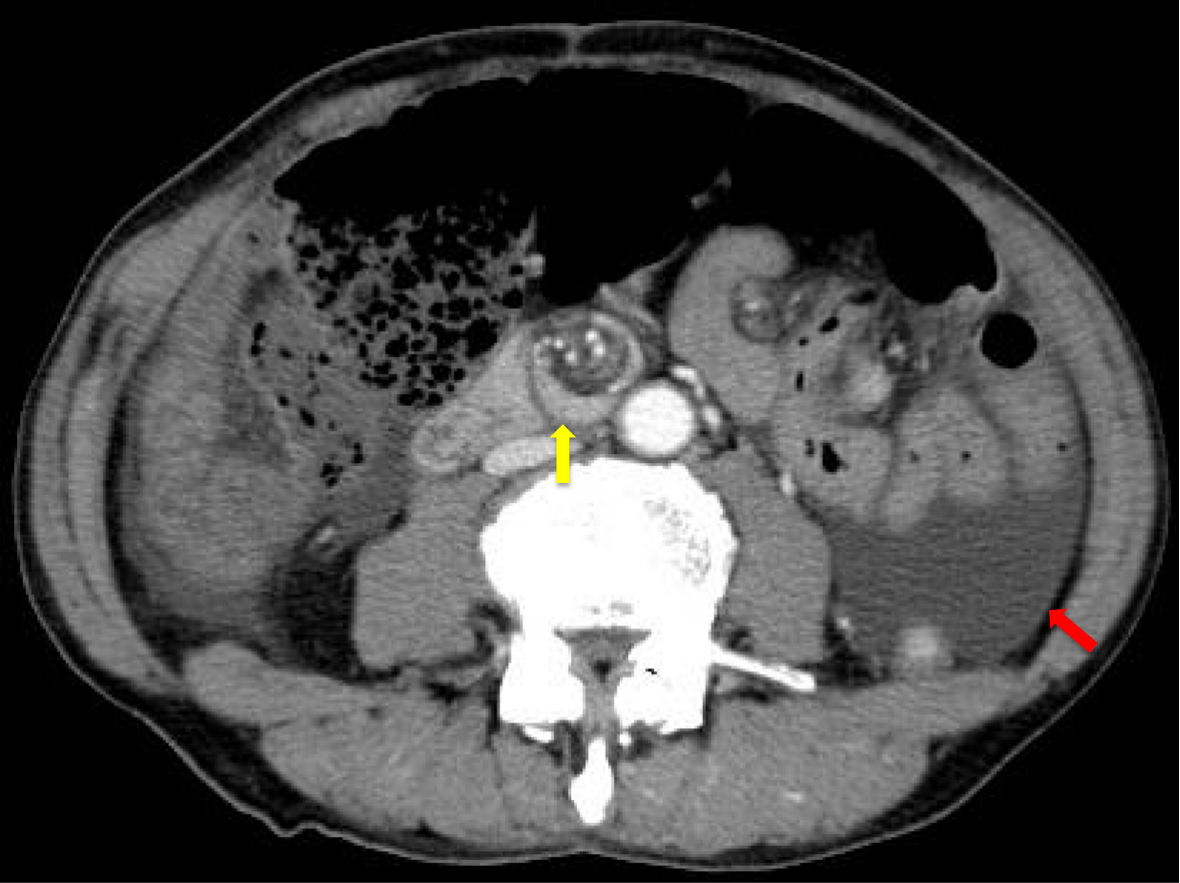
CT scan showing a large amount of ascites (*red arrow*) and whorl-like appearance of the superior mesenteric artery (SMA) and branches with adjacent collapsed loops of small bowel (*yellow arrow*)

Laboratory data were unremarkable except for a white blood cell (WBC) count of 8.7 × 10^9^ cells/L (normal, 4.0–8.0 × 10^9^ cells/L) and a C-reactive protein (CRP) level of 16.2 nmol/L (normal, < 2.9 nmol/L). A contrast-enhanced abdominal CT scan (Fig. 1) showed dilation of the transverse colon, severe ascites, and a whorl-like appearance of the superior mesenteric artery (SMA) and branches wrapped with adjacent mesentery and small bowel loops (the “whirl” or “whorl” sign) [3], which is characteristic of small bowel volvulus. Given these findings, we performed emergency laparotomy.

**Fig. 2:**
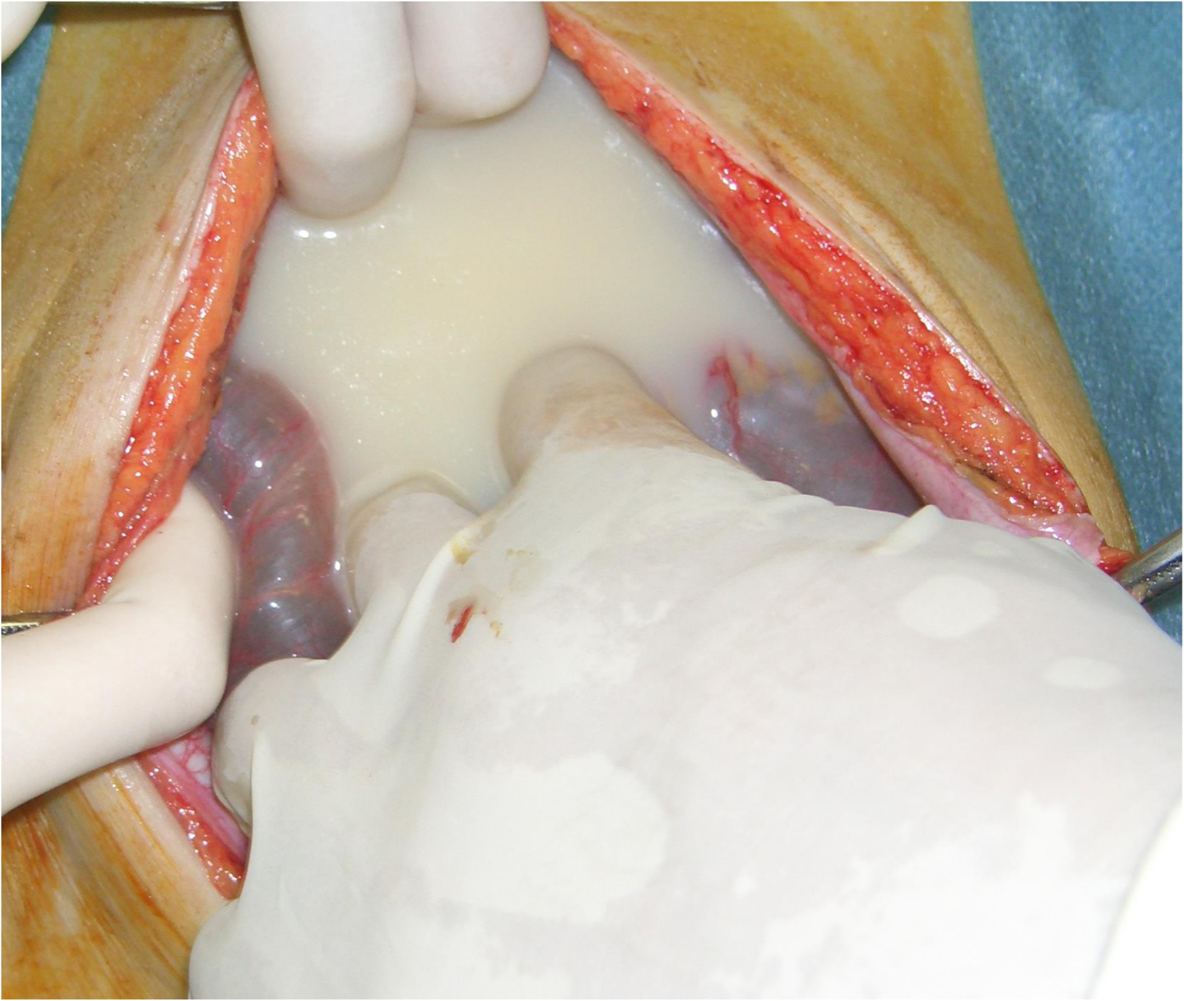
Milky fluid in the peritoneal cavity

**Fig. 3:**
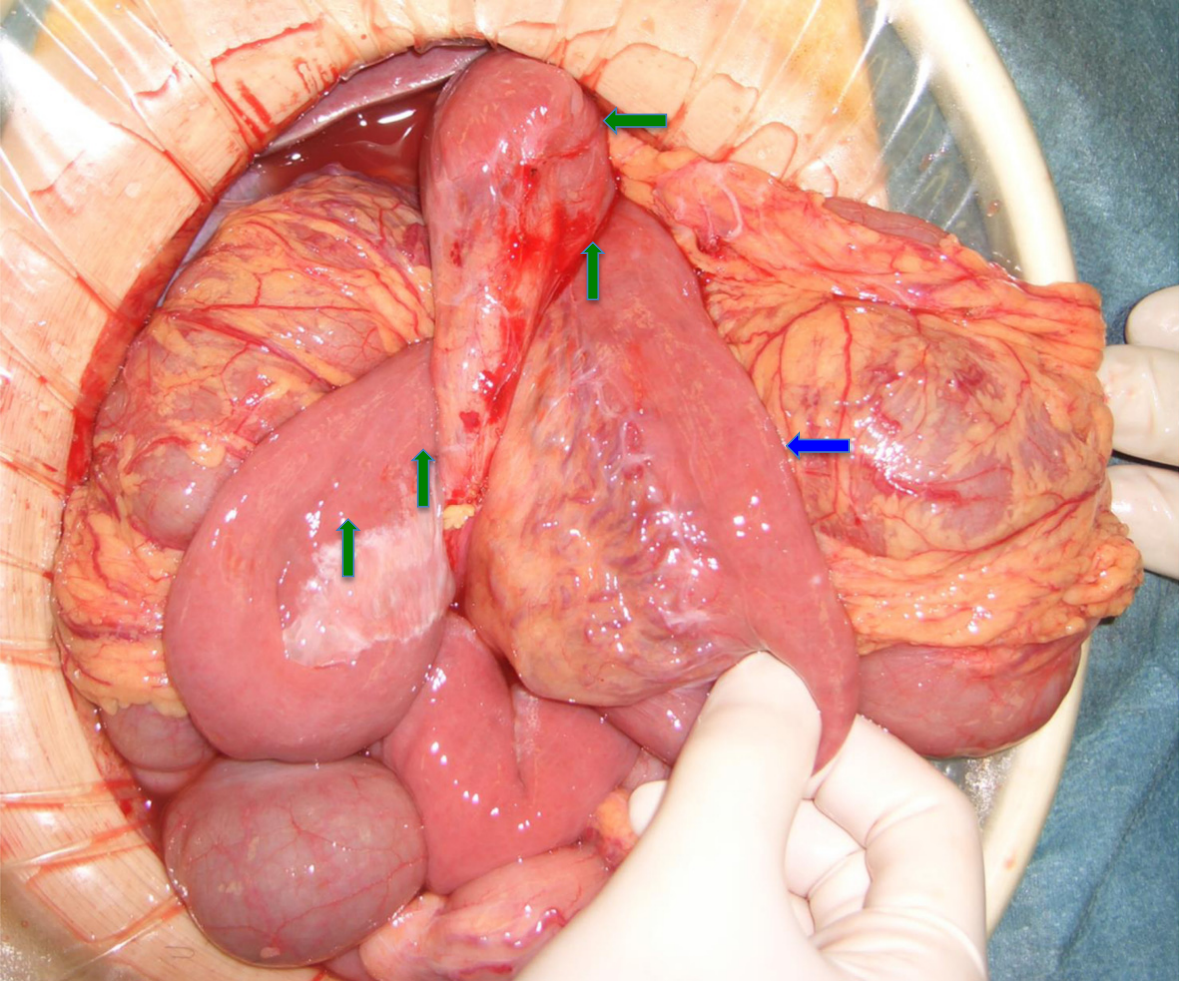
The small bowel passes through the defect between the jejunal mesentery (*blue arrow*) and the mesocolon. The jejunum was rotated 180° counterclockwise in the direction of the long axis (*green arrows*)

Intraoperatively, a massive amount of milky fluid was observed in the peritoneal cavity, and approximately 1 L was removed (Fig. 2). The greater part of the small bowel passed through the defect between the jejunal mesentery and the mesocolon (Petersen’s defect). The small intestine was displaced to the left and was obstructed due to jejunal volvulus (Fig. 3). The jejunum was rotated 180° counterclockwise around its long axis and compressed the transverse colon. The entire small bowel was edematous but viable, with no evidence of a mobile cecum, peritoneal tumor, or perforation. The volvulus and internal hernia were reduced, and the Petersen’s defect was sutured closed. No bowel resection was required. Extensive lavage of the abdominal cavity was performed until the return fluid was clear, and intraperitoneal drains were placed in the rectovesical pouch and under the right hemidiaphragm. The triglyceride (TG) level of the ascitic fluid was 642 mg/dL. The cultures and cytology of the peritoneal fluid were negative for infection and malignancy, respectively.

**Fig. 4:**
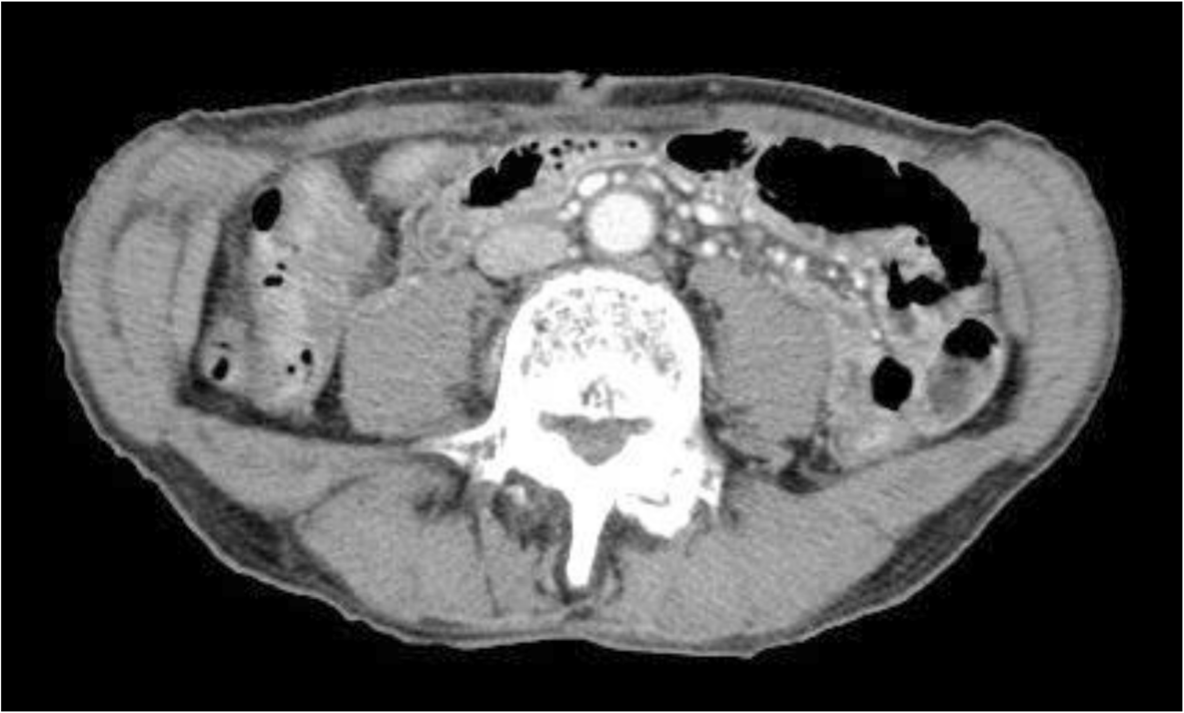
CT performed 10 months after surgery shows no evidence of the recurrence of ascites

**Fig. 5:**
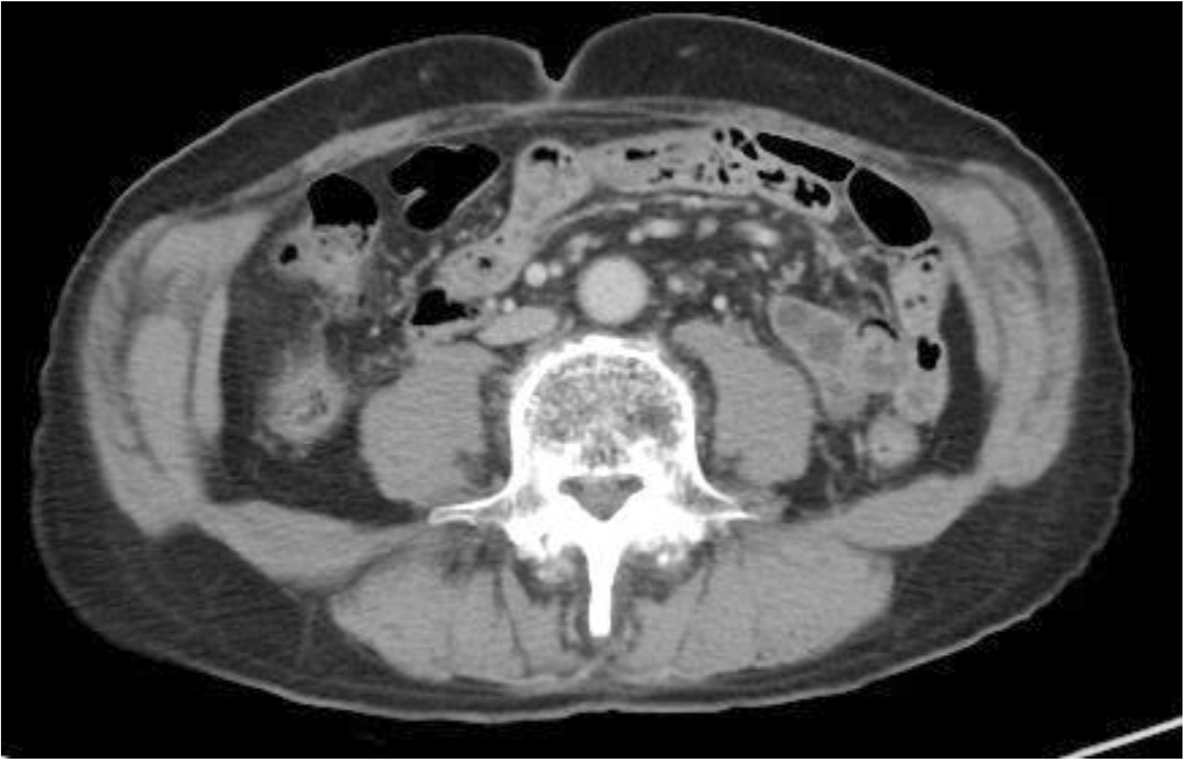
CT performed 28 months after surgery shows increasing subcutaneous fat, indicating improvement in the patient’s health

The patient’s postoperative course was uneventful. The color of the ascites changed from milky to light yellow, and the TG levels decreased to 21 mg/dL on postoperative day 4. The patient was not required to eat a fat-restricted diet during hospitalization and was discharged from the hospital 16 days after surgery. He underwent a physical examination and CT scan in the outpatient department 10 months after discharge from the hospital. There was no evidence of ascites (Fig. 4). He had made favorable progress by the time of the 28-month postoperative follow-up, with evidence of weight gain and no evidence of recurrent ascites (Fig. 5).

### Discussion

Chylous ascites typically develops due to congestion of the lymphatic system. Malignancy—particularly lymphoma—is a relatively common cause of chylous ascites in adults [4]. Metastasis of the malignancy into the lymphatic channels and the resulting occlusions can cause leakage from the dilated subserosal lymphatic system into the peritoneal cavity [5, 6]. However, there have been few reports of chylous ascites associated with intestinal obstruction [7].

In the present case, an abdominal CT scan showed a dilated colon and severe ascites with a whorl-like appearance of the SMA and mesentery, findings generally suggesting small bowel obstruction from volvulus [3, 8], which may be strangulated [1, 9]. High TG levels, typically >200 mg/dL, in the ascitic fluid are critical in defining chylous ascites [10]. Given that our patient had TG levels exceeding 600 mg/dL, the milky fluid was diagnosed as chylous ascites. The CT value of the ascites was 5.0 HU, not suggestive of hemoperitoneum and indistinguishable from that of water [11–13]. Major lymphatic leaks can sometimes be visualized with MR lymphography [14], aiding the diagnosis of chylous ascites. However, minor leaks may be undetectable. After surgery to repair the internal hernia, the TG levels decreased from 642 to 21 mg/dL without any ligation of lymph vessels or dietary restriction. These observations prompted us to diagnose the patient as having chylous ascites associated with small bowel obstruction.

We identified eight case reports of chylous ascites with bowel obstruction in the English language literature (Table 1). Chylous ascites associated with intestinal obstruction was first mentioned by Mackman in 1967, and our present case is the first case report of chylous ascites occurring with a Petersen’s hernia. As in our case, all previous cases were characterized by the presence of small bowel obstruction. Detecting a cause of chylous ascites associated with bowel obstruction is difficult, because these cases are so rare. Of note, however, no previous studies [15–22] reported the requirement for an enterectomy, and their postoperative courses were uneventful.

**Table 1:** Overview of previously reported cases of intestinal obstruction with chylous ascites; no case needed resection

Author [ref number]	Age, sex	Preoperative diagnosis	Cause of obstruction	Surgery	Postoperative course
Mackman [15]	30 months, M	Distended abdomen, unknown etiology	Midgut volvulus	Reduction and cecopexy	Uneventful
Shariff [16]	7 weeks, M	Reducible inguinal hernia	Midgut volvulus	Reduction and Ladd’s procedure	Uneventful
Murugan [17]	44, M	Small bowel volvulus	Small bowel volvulus	Reduction	Uneventful
Seltz [18]	2 weeks, M	Midgut volvulus	Midgut volvulus	Reduction and Ladd’s procedure	Uneventful
Zarroug [19]	12 weeks, M	Midgut volvulus	Midgut volvulus	Reduction and Ladd’s procedure	Uneventful
Hidalgo [20]	40, M	Chronic abdominal pain post laparoscopic Roux-en-Y gastric bypass surgery	Internal hernia of common channel	Reduction	Uneventful
Pengelly [21]	85, F	Acute abdomen	Small bowel volvulus	Reduction	Uneventful
Koh [22]	19, M	Small bowel volvulus	Small bowel volvulus	Reduction	Uneventful

The abdominal CT scan showed that there was an intestinal obstruction and large amounts of ascites. These findings sometimes indicate strangulated obstruction. Our case was interesting in that the physical examination and laboratory tests did not match the severity of the problem as shown on CT. It is difficult to explain this discrepancy, and it is difficult to prove accurately what caused the chylous ascites. This situation is very rare. The absence of bowel ischemia suggests that the arteriovenous system was patent while lymphatic channels were occluded in this case. This vascular patency may explain the discrepancy between the laboratory values and physical findings and the imaging findings, and the fact that no previous cases have reported the requirement for an enterectomy. The obstruction of the lymphatic system resulted in the extravasation of chylous fluid [23]. Pre- and intraoperative evaluation of ascites may be helpful in making decisions concerning therapeutic strategies, because bowel obstruction with chylous ascites may indicate intestinal viability.

## Conclusions

We present a case of chylous ascites associated with intestinal obstruction from volvulus due to a Petersen’s hernia. Detecting the presence of chylous ascites in small bowel obstruction is important, because the therapeutic strategy may differ depending on it. The presence of chylous ascites with intestinal obstruction may represent a particular situation in which the arteriovenous system is patent, and more conservative therapy may be appropriate.

## Consent

Written informed consent was obtained from the patient’s family for the publication of this case report and any accompanying images. A copy of the written consent is available for review from the Editor-in-Chief of this journal.

## Abbreviations

CRP, C-reactive protein; CT, computed tomography; HU, Hounsfield Units; SMA, superior mesenteric artery; TG, triglycerides; WBC, white blood cell count
